# Surface Modifications of cpTi and Ti-6Al‑4V: The Synergistic Potential of PEO-CaP for Optimized Dental Implant Performance

**DOI:** 10.1021/acsomega.5c12236

**Published:** 2026-07-06

**Authors:** Júlia M. T. Teodoro, Maria H. R. Borges, Raphael C. Costa, Jairo M. Cordeiro, Elidiane Rangel, Nilson C. Da Cruz, Carlos A. Fortulan, Bruna E. Nagay, Valentim A. R. Barão

**Affiliations:** † Department of Prosthodontics and Periodontology, Piracicaba Dental School, Universidade Estadual de Campinas (UNICAMP), Piracicaba, São Paulo 13414-903, Brazil; ‡ School of Dentistry, 28132 Alfenas Federal University (UNIFAL-MG), Alfenas, Minas Gerais 37130-001, Brazil; § School of Dentistry, University Center of Associated Teaching Faculties (UNIFAE), São João da Boa Vista, São Paulo 13870-377, Brazil; ∥ Laboratory of Technological Plasmas, Engineering College, Universidade Estadual Paulista (UNESP), Sorocaba, São Paulo 18087-180, Brazil; ⊥ Department of Mechanical Engineering, University of São Paulo (USP), São Carlos, São Paulo 13566-590, Brazil

## Abstract

The surface characteristics of biomaterials are essential to the clinical performance of dental implants, as surface modifications directly affect their mechanical, electrochemical, and biological behavior. This study aimed to identify the most advantageous surface treatment–substrate combination, providing insights that support the development of more durable, bioactive, and clinically effective implant materials. Two surface modification techniques were investigated: sandblasting followed by double acid etching (SLA-like surfaces) and bioactive coatings enriched with calcium (Ca) and phosphorus (P) produced by plasma electrolytic oxidation (PEO-CaP), applied to commercially pure titanium (cpTi) and Ti-6Al-4V alloy. Polished discs of both substrates were divided into six groups (cpTi, Ti6Al4V, cpTi-SLA, Ti6Al4V-SLA, cpTi-PEO, and Ti6Al4V-PEO) and evaluated for their microstructural, mechanical, electrochemical, biological, and bioactive properties. Compared with cpTi, Ti-6Al-4V exhibited higher hardness and mechanical strength, whereas PEO coatings enhanced corrosion resistance by promoting the formation of anatase and rutile crystalline phases. SLA treatment resulted in roughened topographies with pronounced microdepressions, whereas PEO formed porous oxide layers enriched with Ca and P, which enhanced surface bioactivity and provided a more favorable substrate for osteoblastic differentiation compared with SLA. Notably, all tested surfaces induced hydroxyapatite formation, reinforcing their bioactive potential. Collectively, these findings indicate that PEO treatment, particularly on Ti-6Al-4V, provides a synergistic enhancement of mechanical robustness and corrosion resistance, supporting its potential as an effective surface modification strategy for dental implants.

## Graphical abstract

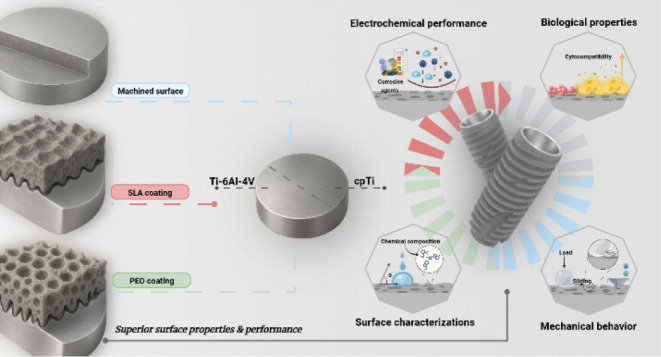

## 1 Introduction

Dental implants represent the most reliable strategy for functional and esthetic oral rehabilitation.^1–3^ In this context, titanium and its alloys, particularly commercially pure titanium (cpTi) and the Ti-6Al-4V alloy, are the most commonly employed materials in implant manufacturing due to their favorable combination of mechanical strength, biocompatibility, and osseointegration capacity.^4–6^ Among these, the superior mechanical properties and corrosion resistance of Ti-6Al-4V can be attributed to its biphasic (α + β) microstructure at room temperature, which provides enhanced tensile strength, hardness, and yield stress.^7–9^ However, despite these advantages, both materials remain susceptible to surface degradation under physiological conditions.^10^ Repetitive mechanical loading and prolonged exposure to corrosive agents, such as saliva, food-derived acids, and oral hygiene products, may compromise the naturally formed passive TiO_2_ layer on titanium surfaces, triggering a cascade of wear and ion release that may affect long-term clinical outcomes.^11,12^

To mitigate these risks and optimize surface properties, a wide range of surface modification strategies has been developed. These approaches aim to enhance the biological behavior of biomedical implants, promote more stable osseointegration, and improve resistance to chemical and mechanical degradation.^13–15^ Among the most widely used techniques are sandblasting followed by acid etching (SLA) and plasma electrolytic oxidation (PEO), which differ in mechanism but share the goal of increasing the biological and electrochemical functionality of implant surfaces. SLA treatments generate microrough topographies with increased surface area via grit blasting and double acid etching,^16,17^ whereas PEO forms porous oxide coatings via high-voltage microdischarges in electrolytic solutions.^18^ PEO also enables the incorporation of bioactive elements, such as calcium (Ca) and phosphorus (P),^19,20^ and the formation of crystalline phases of TiO_2_ (anatase and rutile), which are associated with enhanced biological and anticorrosion properties.^21–23^ However, although both techniques show promise, their effects may vary significantly depending on the underlying metallic substrate.^24–26^

It is essential to note that, despite the widespread reporting of the individual benefits of SLA and PEO treatments, most investigations focus on a single titanium-based substrate, which limits our understanding of how, and whether, the metal substrate composition affects the performance of each treatment. Comparative studies between cpTi and Ti-6Al-4V tend to emphasize bulk mechanical performance.^27,28^ Yet, differences in surface response may directly affect clinical performance, influencing not only biological processes but also the long-term stability of implant-supported rehabilitations under functional loading.^14,25,29^ In addition, studies integrating corrosion resistance, tribological behavior, and biological performance under comparable conditions are still scarce, hindering a more definitive understanding of the clinical relevance of these surface modification techniques. Conflicting findings in the literature further highlight the lack of consensus regarding the most suitable surface treatment–substrate combination. For these reasons, a more holistic understanding of how each treatment alters the material at multiple levels is essential to guide evidence-based decisions in implant design and material selection.

In light of these challenges and motivated by the need for a more integrative understanding of how different surface treatments interact with distinct titanium-based substrates, the present study aimed to perform a systematic comparative analysis of cpTi and Ti-6Al-4V substrates subjected to two distinct surface treatments: SLA-like and PEO enriched with Ca and P (PEO-CaP). A comprehensive experimental approach was conducted to assess morphological and microstructural features, mechanical properties, corrosion resistance, and biological performance. By correlating surface features with functional outcomes, this study seeks to clarify the extent to which each surface treatment influences functional performance depending on the substrate, providing insights that support the development of more durable, bioactive, and clinically effective implant materials.

## 2 Materials and Methods

### 2.1 Experimental Design

Discs (Ø = 10 mm × 1 mm) of commercially pure titanium (cpTi) and titanium–aluminum–vanadium alloy (Ti-6Al-4V) were polished and randomly assigned to six groups. Machined cpTi and Ti-6Al-4V served as controls, whereas the experimental groups consisted of surfaces treated by SLA and PEO/Ca–P: cpTi-SLA, cpTi-PEO, Ti-6Al-4V-SLA, and Ti-6Al-4V-PEO. Physical, chemical, mechanical, electrochemical, and biological tests were performed to investigate the effects of the two surface modifications (SLA and PEO) on the different metallic substrates (cpTi and Ti-6Al-4V). A schematic representation of the experimental design is provided in Figure 1.

**Figure 1:**
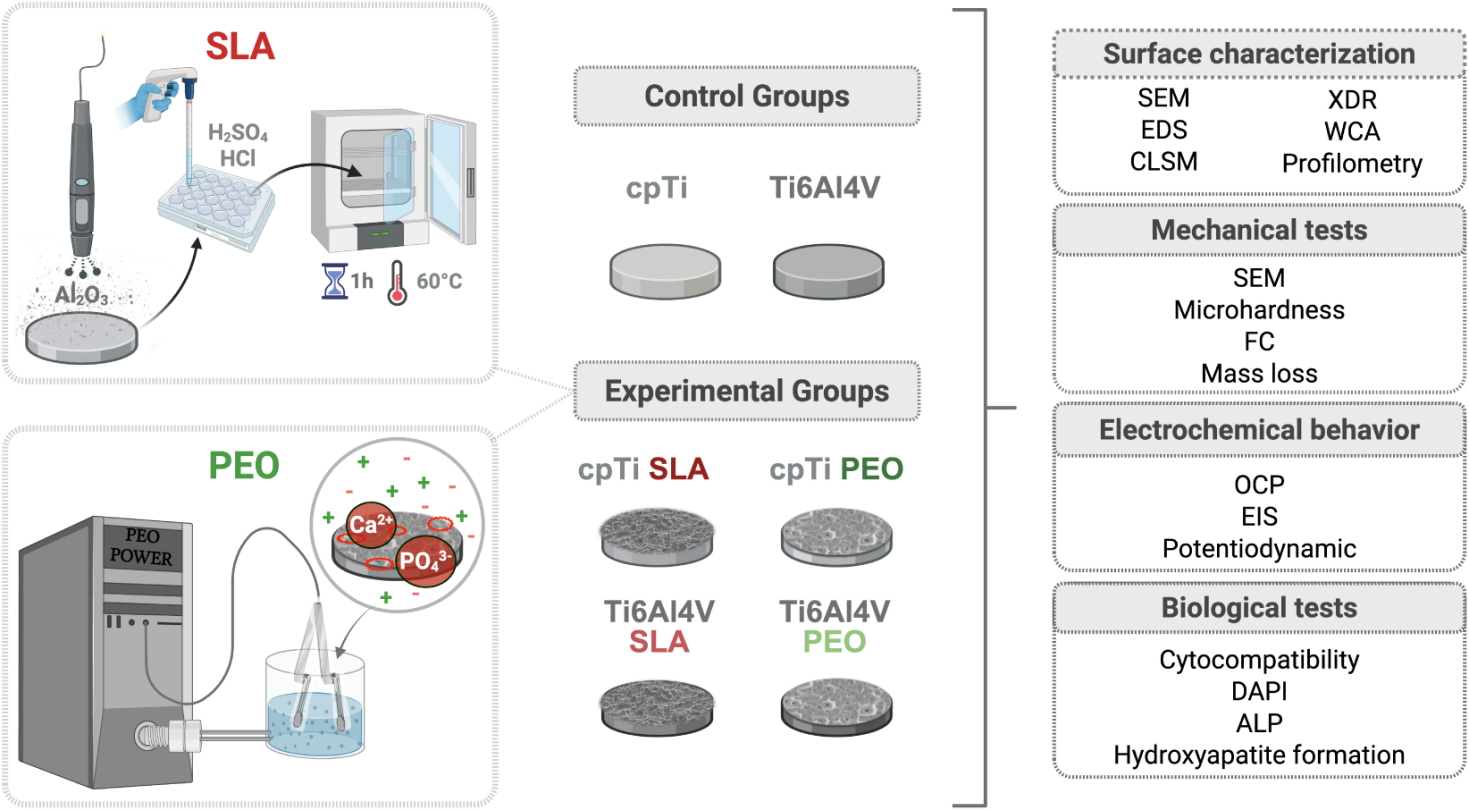
Schematic diagram of the experimental design. SLA = sandblasting followed by acid etching; PEO = plasma electrolytic oxidation; SEM = scanning electron microscopy; EDS = energy-dispersive X-ray spectroscopy; CLSM = confocal laser scanning microscopy; XRD = X-ray diffraction; WCA = water contact angle; FC = coefficient of friction; OCP = open-circuit potential; EIS = electrochemical impedance spectroscopy; ALP = alkaline phosphatase activity. Created with BioRender.com (License number: NW29LSEPNN).

### 2.2 Surface Preparation

Grade 2 cpTi and grade 5 Ti-6Al-4V discs (Realum, São Paulo, Brazil) were sequentially polished with #320 and #400 grit papers (Carbimet 2; Buehler, USA) using an automatic polishing machine (EcoMet 300 Pro with AutoMet 250; Buehler) under constant irrigation and speed. Samples were then ultrasonically cleaned in an enzymatic detergent solution, followed by deionized water and 70% propanol (three 10 min cycles), and then dried with hot air. Polished cpTi and Ti-6Al-4V specimens served as control groups.^30–32^

For SLA surface preparation, 150 μm Al_2_O_3_ particles (99.7% purity; Polidental Indústria Comércio Ltd., Cotia, Brazil) were blasted onto the specimens at a distance of 50 mm, at a 90° angle, under a pressure of 0.45 MPa for 30 s. Although the alumina used in this study does not strictly replicate industrial processing conditions, its particle size and characteristics were selected to be representative of those commonly reported in the literature for SLA-like surface treatments.^16,17^ The samples were then immersed in an acid solution containing 18% (v/v) HCl (99.9% purity; Sigma-Aldrich, St. Louis, MO, USA) and 49% (v/v) H_2_SO_4_ (99.9% purity; Sigma-Aldrich) at 60 °C for 1 h to perform the chemical treatment.^33,34^ Following this process, the specimens were rinsed under running water and air-dried at room temperature.

The PEO surface treatment was performed using a pulsed direct current (DC) power supply (Plasma Technology Ltd., Kowloon, Hong Kong). The electrolyte composition and PEO parameters were selected based on prior reports that demonstrated optimal performance in dental implant applications. An electrolyte containing 0.3 M calcium acetate and 0.02 M sodium glycerophosphate was used to promote the incorporation of Ca and P species into the oxide layer. PEO treatment was performed at a pulsed voltage of 290 V, a frequency of 250 Hz, and a duty cycle of 60% for 10 min, as these conditions have been shown to yield coatings with enhanced surface hardness, bioactivity, protein adsorption, and corrosion resistance.^21,32^ The process consisted of oxidizing the specimens, serving as the anode, immersed in an electrolytic solution containing 0.3 M calcium acetate [Ca­(CH_3_CO_2_)_2_] (99% purity; Dinâmica Ltd., Diadema, SP, Brazil) and 0.02 M sodium glycerophosphate [C_3_H_7_Na_2_O_6_P] (99% purity; Dinâmica Ltd.) inside a stainless steel tank equipped with a cooling system (23.0 ± 1.5 °C), which served as the cathode. Finally, the PEO-treated discs were rinsed with deionized water and air-dried for 24 h.^35^

### 2.3 Structural Morphology/Topography and Chemical Composition

Surface morphology was examined using Scanning Electron Microscopy (SEM; JEOL JSM-601LA, Peabody, MA, USA) operated at 15.0 kV. Additionally, surface topography was assessed using noncontact three-dimensional Confocal Laser Scanning Microscopy (CLSM; VK-X200 series, Keyence, Osaka, Japan). The acquired images were processed and analyzed using the VK-Analyzer software (version 3.3.0.0; Keyence, Osaka, Japan).^34^

### 2.4 Chemical Composition and Crystalline Phases of the Treatments

Energy-dispersive X-ray spectroscopy (EDS; Bruker, Germany) was used to analyze the elemental composition of the specimens with a resolution of approximately 1 μm^3^. Results were reported both quantitatively, as elemental proportions, and qualitatively, through elemental distribution maps.^35^ The crystalline phases present on the treated surfaces were identified by X-ray diffraction (XRD; Panalytical X’Pert3 Powder, UK) using Cu Kα radiation (λ = 1.5418 Å) operated at 45 kV and 40 mA, with a continuous scan rate of 0.02°/s over a 2 h run.^36^

### 2.5 Surface Roughness and Wettability

Surface roughness was assessed using a contact profilometer (Dektak 150-d; Veeco, Plainview, NY, USA). Three linear measurements were taken at the center, left, and right regions of each specimen under the following conditions: cutoff length of 0.25 mm, scanning speed of 0.05 mm/s, and measurement time of 12 s.^4^ Wettability and surface free energy were determined using a goniometer (Ramé-Hart 10000; Ramé-Hart Instrument Co., Succasunna, NJ, USA) via the sessile-drop method (10 μL droplet). Contact angles were measured with deionized water (polar component) and 1-bromonaphthalene (nonpolar component), and the results were analyzed using dedicated software (DROPimage Standard, Ramé-Hart Instrument Co.) according to Young’s model.^20,37^

### 2.6 Mechanical Properties

#### 2.6.1 Vickers Hardness

Vickers microhardness was measured using a hardness tester (HMV-2, Shimadzu Corporation, Kyoto, Japan). The hardness values were calculated using the formula HV = 1.8544 P/d^2^, where P is the applied load and d is the average length of the indentation diagonals. A load of 0.5 kgf was used for 15 s, with four randomly distributed indentations performed on each specimen.^38^

#### 2.6.2 Tribological Test

The friction coefficient was determined using a custom-built pin-on-disk tribometer (Faculty of Mechanical Engineering, University of São Paulo, São Carlos, Brazil).^39^ Friction evolution and average coefficient values were recorded with LabView software (National Instruments, São Paulo, Brazil).^36^ Mass loss (mg) was calculated by weighing each disc before and after testing using an analytical balance (AUY-UNIBLOC, Shimadzu Corporation, Kyoto, Japan).^34^ Wear scars were analyzed by SEM (JEOL JSM-6010 LA, Peabody, MA, USA). Wear area was determined using an optical microscope (VMM-100-BT; Walter UHL, Asslar, Germany) coupled with a digital camera (KC-512NT; Kodo BR Eletronica Ltd., São Paulo, Brazil) and an analyzer unit (QC 220-HH Quadra Check 200; Metronics Inc., Bedford, MA, USA).^31,32^ Detailed test parameters are described in the Supporting Information.

### 2.7 Electrochemical Behavior

Electrochemical tests were performed to assess the corrosion stability of the surfaces in simulated body fluid (SBF; 10 mL; 37 °C). A three-electrode cell, connected to a potentiostat (Interface 1000, Gamry Instruments, Warminster, PA, USA), was used to perform open-circuit potential (OCP) measurements, electrochemical impedance spectroscopy (EIS), and potentiodynamic polarization.^40–43^ Further details are provided in the Supporting Information.

### 2.8 Biological Performance

#### 2.8.1 Preosteoblastic Cell Culture and Cell Viability

To evaluate cytocompatibility, mouse calvaria-derived preosteoblastic MC3T3-E1 cells (ATCC CRL-2594; Banco de Células do Rio de Janeiro) were cultured directly on the sample surfaces in α-MEM (Gibco, Life Technologies, USA) supplemented with 10% fetal bovine serum (FBS; Gibco, Grand Island, NY, USA), 100 U/mL penicillin, and 100 μg/mL streptomycin, under standard conditions (37 °C, 5% CO_2_).^32^ Later, cell metabolic activity was assessed using the AlamarBlue assay on days 3 and 7.^44,45^ Specific testing conditions are outlined in the Supporting Information.

#### 2.8.2 Cell Adhesion and Proliferation

Additionally, cell density was assessed by fluorescence-based nuclear staining at days 3 and 7. For this purpose, the samples were incubated with DAPI (4′,6-diamidino-2-phenylindole) prepared in phosphate-buffered saline (PBS) at a concentration of 1 μL/mL.^33^ After fixation, the cell membranes were permeabilized with 1% Triton for 30 min, followed by DAPI staining (5 mg/mL; Sigma-Aldrich) for 10 min. The discs were then rinsed with PBS and protected from light until analysis. Images were captured using a fluorescence microscope (Leica DM IRB, Japan), and nuclei density (nuclei/mm^2^) was quantified in ImageJ software.

#### 2.8.3 Osteoblastic Differentiation by Alkaline Phosphatase (ALP) Activity

ALP activity, as an early marker of osteoblastic differentiation, was quantified using a colorimetric assay kit (BioAssays Systems) according to the manufacturer’s instructions. The analysis was performed after 7 days of incubation. Briefly, cultures were washed with PBS and lysed in PBS containing 0.2% Triton X-100 overnight at room temperature with agitation. A 10 mM p-nitrophenyl phosphate substrate solution was then added, and absorbance was measured at 405 nm using a microplate reader (Infinite 200 Pro, TECAN). Samples were analyzed in triplicate, and ALP activity was normalized to the corresponding metabolic activity values.^33^

#### 2.8.4 Hydroxyapatite Formation

Surface bioactivity was assessed by measuring hydroxyapatite (HAp) deposition. Samples were immersed in custom-made plastic containers filled with SBF and maintained under static conditions at 37 ± 1 °C for 21 days. The SBF volume was adjusted based on the exposed surface area and renewed daily with a freshly prepared solution. After incubation, disks were rinsed with distilled water, air-dried in a desiccator for 24 h, and analyzed by XRD and SEM to confirm HAp formation and morphology.^32^

### 2.9 Statistical Analysis

Data normality was verified using the Shapiro–Wilk test. A two-way ANOVA was applied to evaluate the differences between materials (factor 1, two levels) and surface treatments (factor 2, three levels). When significant effects were detected, Tukey’s HSD and Dunn’s tests were used for post hoc multiple comparisons. A significance level of *p* < 0.05 was adopted for all analyses.

## 3 Results

### 3.1 Surface Morphology, Composition, and Properties

Morphological analysis by SEM revealed notable differences across the groups. Specimens from the control groups (polished samples) exhibited smooth, homogeneous surfaces with longitudinal grooves resulting from polishing (Figure 2A). Notably, cpTi displayed more pronounced peak (orange tones) and valley (green-to-blue tones) regions compared with the Ti-6Al-4V alloy, suggesting a surface with higher topographical variation (Figure 2B). Samples subjected to the SLA-type surface treatment showed a rough morphology with microdepressions, sharp edges, and micropits, typical of surfaces chemically etched with acids. In contrast, the PEO treatment resulted in the formation of a uniform porous layer with micropores homogeneously distributed across the entire surface. Interestingly, cpTi surfaces exhibited larger pore diameters, whereas Ti-6Al-4V showed smaller pores. Compared within the same treatment, cpTi samples exhibited more pronounced topographies than Ti-6Al-4V (Figure 2B) in both the SLA and PEO groups, indicating a greater amplitude of peaks and valleys, likely attributable to the structural and physical properties of the metallic substrate.

**Figure 2:**
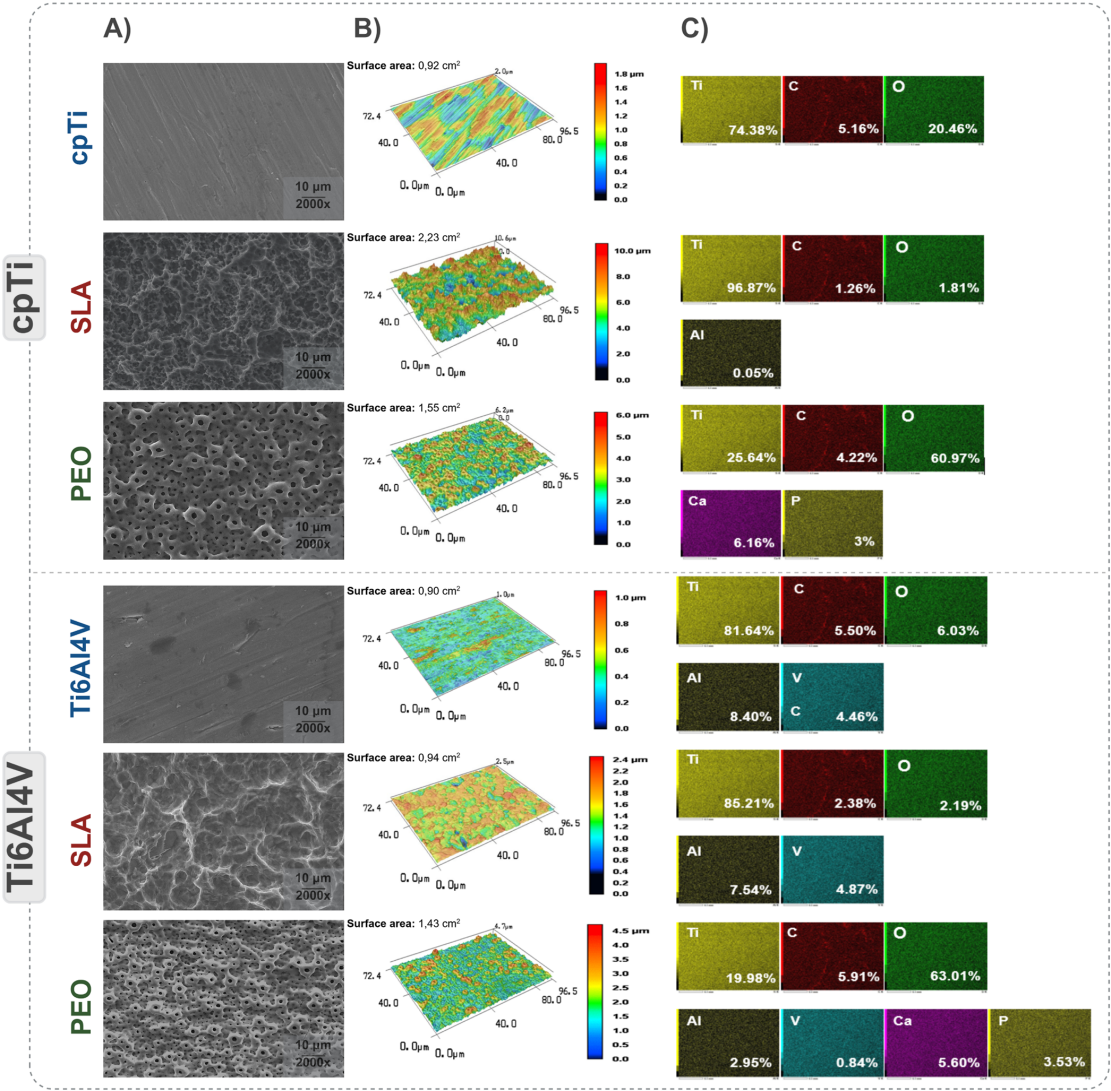
Surface morphology and chemical composition of the groups (*n* = 3/group). (A) SEM micrographs, top view (2000×), (B) three-dimensional CLSM images (50×), and (C) EDS chemical mapping with elemental concentrations (wt %) on the surfaces.

The EDS mapping (Figure 2C) revealed the presence of major elements, including titanium (Ti), oxygen (O), and carbon (C), in all analyzed groups. In Ti-6Al-4V specimens, aluminum (Al) and vanadium (V) were also detected, confirming the characteristic composition of this alloy. In addition, a detectable amount of aluminum was observed on the SLA-treated cpTi surfaces, attributable to residual particles from the sandblasting/acid-etching procedure. A lower aluminum signal was observed on the corresponding PEO-treated Ti-6Al-4V specimens, suggesting adequate coverage of this element by the oxide layer formed during anodization. Regarding vanadium, quantitative EDS analysis showed a marked decrease in its surface content after PEO treatment compared with the polished and SLA-treated Ti-6Al-4V specimens (4.46 at. % V for polished, 4.87 at. % V for SLA, and 0.84 at. % V for PEO). Although the elemental maps may suggest a relatively uniform V distribution on the PEO-treated surface, this result should be interpreted with caution. The PEO coating exhibits a porous morphology with open pores and microdischarge channels, allowing the SEM–EDS interaction volume to partially probe the underlying Ti-6Al-4V substrate rather than being confined to a dense oxide layer. Consequently, residual V from the substrate can still be detected and appear homogeneously distributed in the maps, despite its limited incorporation into the outer oxide. The substantial reduction in V content measured by quantitative EDS confirms that, although detectable, vanadium is not significantly incorporated into the PEO-derived oxide layer.

Furthermore, surfaces treated with PEO exhibited calcium (Ca) and phosphorus (P), confirming the successful incorporation of these elements. Moreover, a higher oxygen content was detected, consistent with oxide formation during anodization, reinforcing the effectiveness of the electrochemical treatment in promoting surface oxidation.

Regarding the crystalline composition, XRD patterns (Figure 3A) revealed the presence of the metallic Ti phase in all analyzed surfaces. In contrast, PEO samples presented a combination of TiO_2_ crystalline phases, with characteristic peaks of anatase (∼25°) and rutile (∼27°). SLA-treated surfaces exhibited peaks attributed to titanium hydride (∼43°), indicating the formation of this phase during treatment.

**Figure 3:**
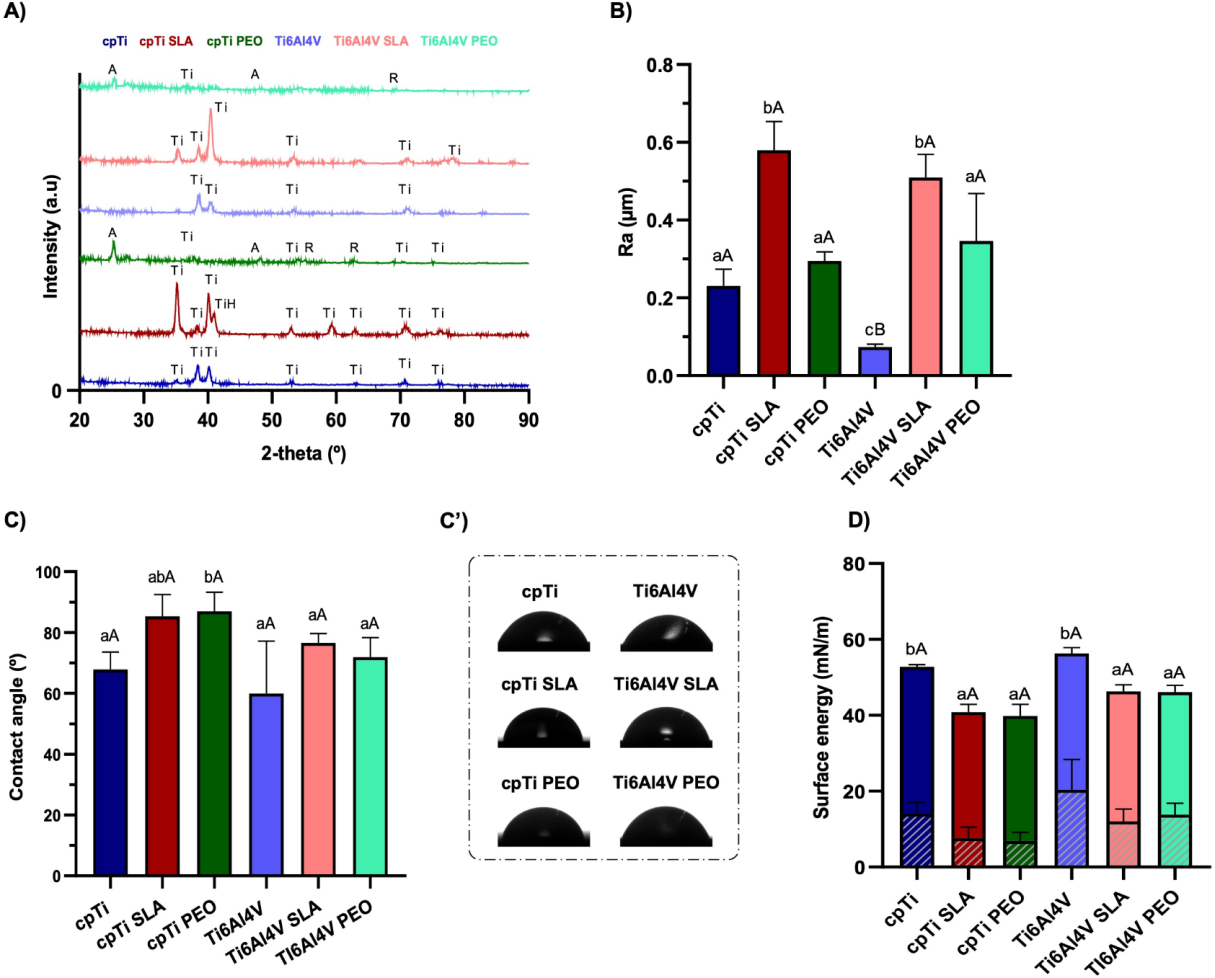
Physicochemical characterization of control and experimental groups (*n* = 5/group). (A) XRD patterns of surfaces showing crystalline phase peaks. (B) Surface roughness parameters [Ra = arithmetic roughness] obtained by profilometry. (C) Water contact angle and (C’) representative images of contact angles on surfaces. (D) Surface free energy. Different lowercase letters indicate statistically significant differences among surface treatments. Different uppercase letters indicate statistically significant differences between materials (*p* < 0.05, Tukey’s test).

Regarding surface roughness (Figure 3B), the groups subjected to SLA treatment exhibited significantly higher mean roughness (Ra) values compared to the other experimental groups (*p* < 0.05). This increase is directly associated with the morphological modifications induced by acid etching, which enhance surface irregularities by forming microdepressions and sharp edges. The machined samples also showed statistically significant differences between materials (*p* < 0.05), with the Ti-6Al-4V alloy exhibiting lower roughness than cpTi, consistent with the topographical findings observed by SEM.

The PEO and SLA surface treatments increased the contact angle of the specimens (Figure 3C), with more pronounced values observed in the groups treated on cpTi, where the increase was statistically significant (*p* < 0.05), along with a lower surface free energy (Figure 3D) compared with the control groups (*p* < 0.05). However, the substrate type did not account for substantial variations in surface wettability, as both materials exhibited similar responses to the applied treatments.

### 3.2 Mechanical Properties

The tribological outcomes (Figure 4) revealed significant differences in wear behavior between cpTi and Ti-6Al-4V, as well as among the different applied surface treatments. SEM analysis and visual inspection of the wear tracks (Figure 4A) showed distinct wear areas (WA) across both substrates and treatments. The cpTi, cpTi SLA, and cpTi PEO groups presented mean wear areas of 0.50 μm^2^, 0.33 μm^2^, and 0.18 μm^2^, respectively, whereas the Ti-6Al-4V, Ti-6Al-4V SLA, and Ti-6Al-4V PEO groups exhibited mean values of 0.54 μm^2^, 0.24 μm^2^, and 0.14 μm^2^, respectively. These results demonstrate a consistent reduction in wear area following surface treatment, particularly with PEO, across both metallic substrates.

**Figure 4:**
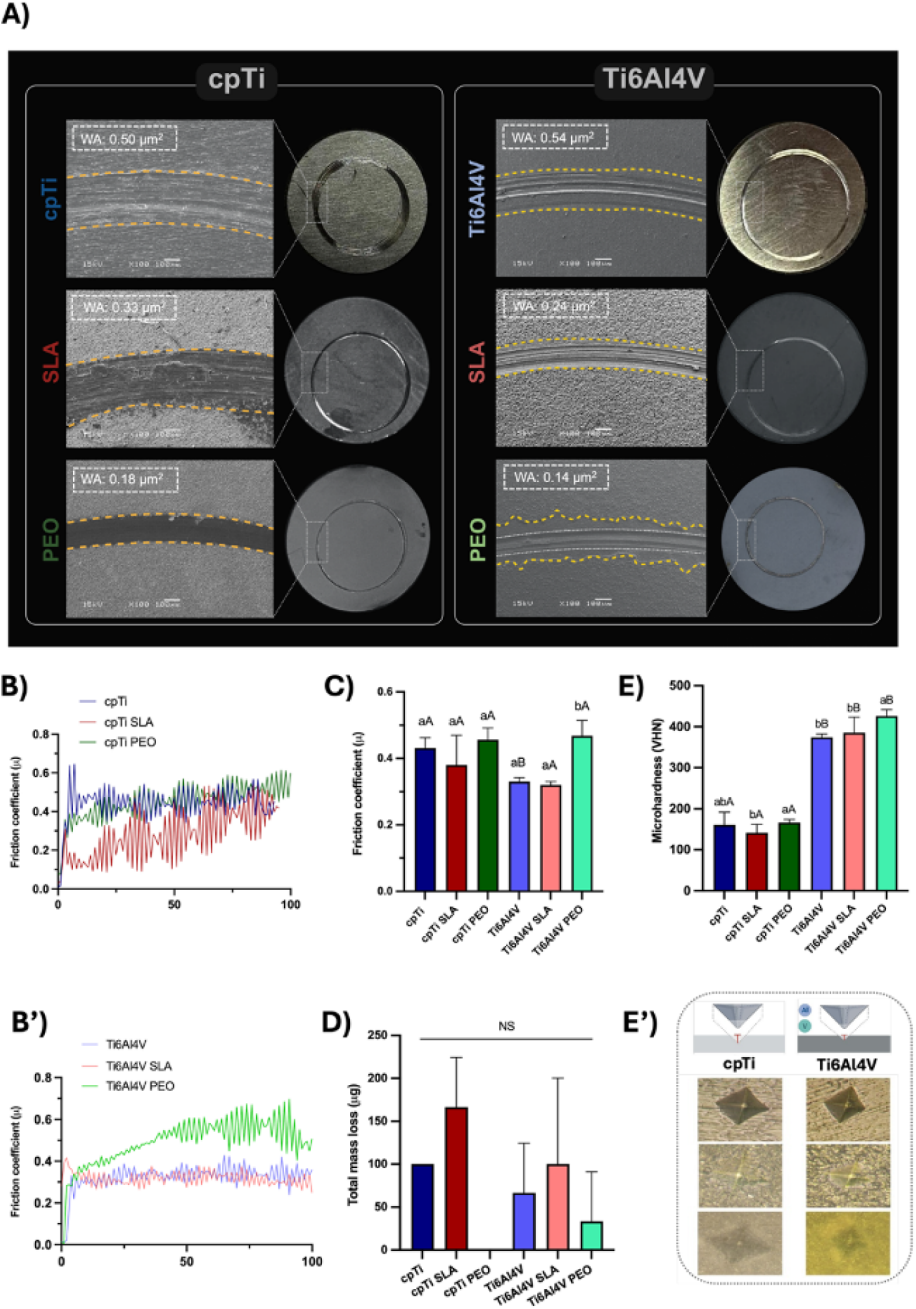
Tribological behavior of the groups (*n* = 5/group). (A) Disc surfaces (right) and SEM images (left; 100× magnification) showing the wear tracks after tribological testing (WA = wear area in μm^2^). (B, B’) Tribological behavior during the test. (C) Mean coefficient of friction (μ) during sliding. (D) Total mass loss (μg) of the samples before and after tribological wear. (E, E’) Vickers hardness and diamond-shaped indentation image for each surface. Different lowercase letters indicate statistically significant differences among surface treatments. Different uppercase letters indicate statistically significant differences between materials (*p* < 0.05, Tukey’s test). Schematic representation of the indentation produced by the durometer. Created with BioRender.com (License number: GW23RMY6O1).

The evolution profile of the coefficient of friction (Figure 4B,B’) displayed distinct patterns during sliding, reflecting the dynamic tribological behavior of the different groups. Regarding the mean coefficient of friction (Figure 4C), a slight increase was observed in the cpTi PEO group compared with the cpTi control and cpTi SLA groups; however, this difference was not statistically significant (*p* > 0.05). In contrast, comparing the cpTi and Ti-6Al-4V control groups revealed a significant difference (*p* < 0.05), with the Ti-6Al-4V alloy exhibiting a lower coefficient of friction. Assessing treatment effects on Ti-6Al-4V, the PEO group exhibited a significantly higher coefficient of friction compared to the Ti-6Al-4V control and Ti-6Al-4V SLA groups. This outcome may be attributed to the alloy’s chemical composition and the increased hardness of the PEO group, as supported by the microhardness results described above.

After the tribological test, the specimens were weighed, and the analysis showed that the mass loss (Figure 4D) varied among the groups; however, these differences did not reach statistical significance. These findings suggest that, although the PEO treatment enhances surface wear resistance, it may also influence the dynamic friction behavior of the surface, driven by topographical modifications and the presence of oxides formed during the process.

Finally, the Vickers hardness results (Figure 4E,E’) showed that the Ti-6Al-4V alloy exhibited significantly higher values than cpTi across all analyzed groups (*p* < 0.05), reflecting the alloy’s intrinsically greater mechanical strength. In the cpTi groups, the slight decrease in mean hardness observed for cpTi SLA compared with untreated cpTi was not statistically significant (*p* > 0.05). Additionally, the PEO treatment yielded higher hardness values compared with the SLA treatment, regardless of the metallic substrate (*p* < 0.05). This increase may be associated with the formation of a ceramic layer during the plasma electrolytic process, which enhances surface hardness and corroborates the tribological results.

### 3.3 Electrochemical Performance

The electrochemical behavior of the surface was evaluated by OCP, EIS, and potentiodynamic tests. The OCP evolution over 1 h (Figure 5A) revealed that the PEO-treated groups exhibited more noble electrochemical behavior compared with the other groups. In particular, the cpTi-PEO group exhibited the highest positive values, suggesting a more stable surface with greater corrosion resistance. All groups, regardless of treatment or substrate, formed a stable passive layer after 1 h of immersion in SBF, demonstrating the materials’ ability to form a protective oxide film under physiological conditions.

**Figure 5:**
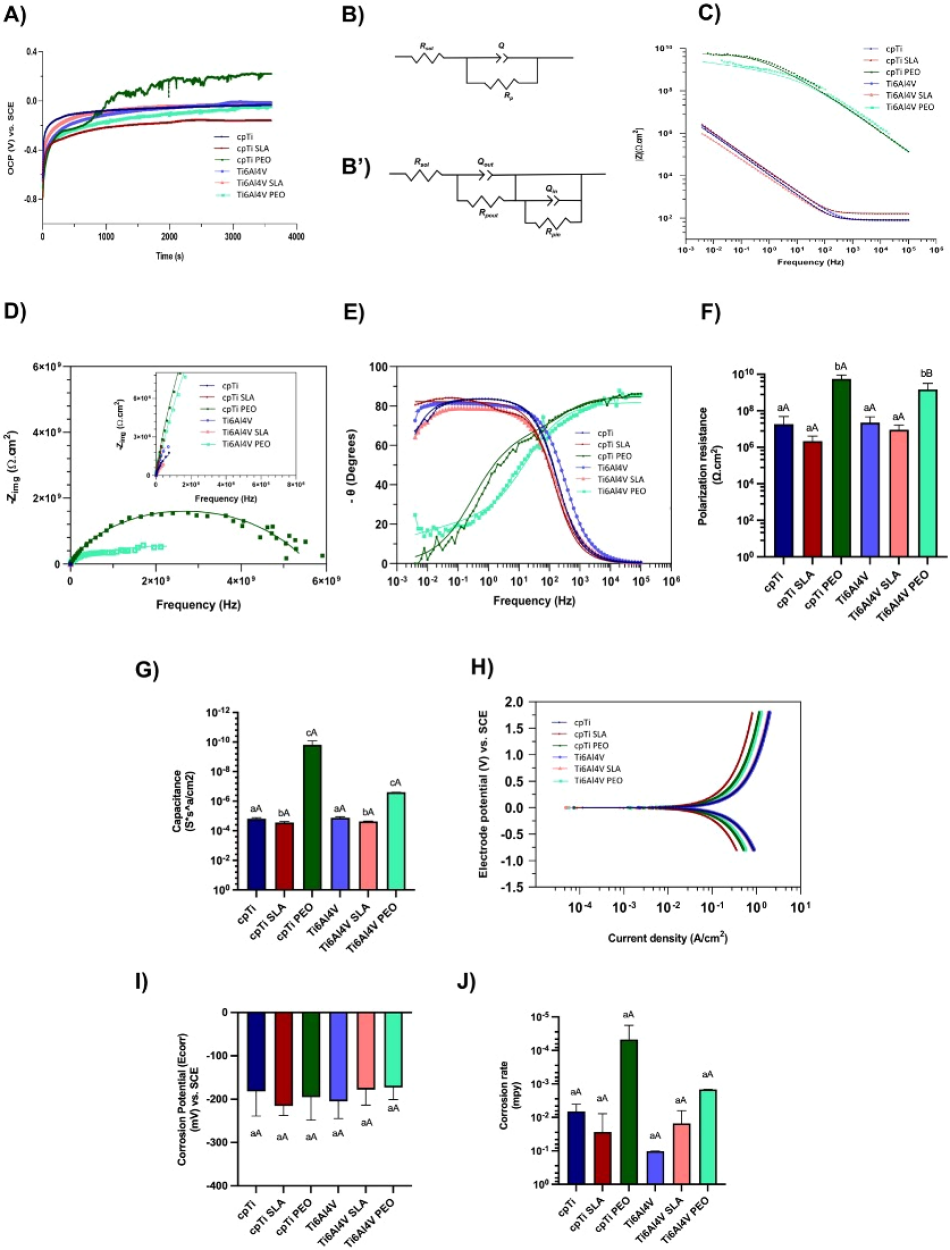
Electrochemical outcomes (*n* = 5/group). (A) Evolution of the open-circuit potential of surfaces over time in SBF. (B) Equivalent electrical circuits used to fit EIS data. (C) Representative impedance (|Z|), (D) Nyquist, and (E) phase angle plots. (F) Polarization resistance and (G) capacitance. (H) Representative potentiodynamic polarization curves. (I) Corrosion potential (E_corr_) and (J) corrosion rate values were obtained. Different lowercase letters indicate statistically significant differences among surface treatments. Different uppercase letters indicate statistically significant differences between materials (*p* < 0.05, Tukey’s test).

EIS was used to investigate the corrosion resistance and dielectric response of the surfaces. Equivalent electrical circuits were applied to model the experimental data (Figure 5B,B’). In the control groups, a single-layer circuit was used for data fitting, indicating a single dielectric system. In contrast, for the SLA and PEO groups, due to the formation of a bilayer structure composed of an outer and inner layer, it was necessary to employ a modified circuit containing two Rp (polarization resistance) and Q (constant phase element, CPE) pairs. The chi-square (χ²) values obtained were of the order of 10^–3^, indicating excellent agreement between the experimental and simulated EIS data. In the impedance–frequency analysis (Figure 5C), the highest impedance values were observed at low frequencies for the PEO groups. The Nyquist diagram (Figure 5D) illustrated the relationship between the real (Zreal) and imaginary (Zimag) impedance components, in which the PEO-treated groups exhibited semicircular arcs with larger diameters and magnitudes, indicating higher charge-transfer resistance. The slight differences observed among the PEO curves (green lines in Figure 5C–D) can thus be attributed to subtle substrate-dependent variations in coating morphology and dielectric behavior (cpTi vs Ti-6Al-4V), rather than to fundamentally different corrosion mechanisms. Notably, in all EIS representations, both PEO groups consistently occupied the region of highest impedance and largest arc diameter, clearly separated from the control and SLA groups, underscoring that the presence of the PEO–CaP layer is the dominant factor controlling the improved electrochemical response. This characteristic was most evident in the cpTi-PEO group, which displayed the largest arc amplitude, necessitating an enlarged plot for complete visualization. Phase angle evaluation (Figure 5E) revealed greater amplitude for the PEO groups, indicating a more pronounced capacitive response and, consequently, a more effective dielectric barrier. Among them, the cpTi-PEO group exhibited slightly higher phase angles than the Ti-6Al-4V-PEO group, suggesting slightly superior electrochemical stability. Nonetheless, both PEO-treated conditions exhibited markedly more capacitive and protective behavior than their respective non-PEO counterparts, regardless of whether the underlying substrate was cpTi or Ti-6Al-4V. Additionally, the cpTi-PEO group exhibited the highest Rp (5.57 × 10^09^ MΩ·cm^2^), significantly higher than all other groups (*p* < 0.05) (Figure 5F). In contrast, the SLA-treated groups showed the lowest R_p_ values, and the PEO-treated surfaces presented lower capacitance values (1.52 × 10^–10^ S·s^a·cm^–2^ for cpTi-PEO and 2.46 × 10^–07^ S·s^a·cm^–2^ for Ti-6Al-4V-PEO) (Figure 5G), consistent with the larger surface area generated by the micropores in the outer layer.

Interestingly, despite the differences observed in the impedance analysis described above, the cyclic polarization test did not reveal variations in behavior among the groups (Figure 5H). The Tafel curves exhibited wide passivation regions across all groups, with stable current density maintained as the applied potential increased. No discontinuities associated with depassivation or repassivation phenomena were observed, suggesting stable electrochemical behavior in a physiological environment. Consistent with this behavior, no statistically significant differences were observed in corrosion potential or corrosion rate (Figure 5I and J).

### 3.4 Cytocompatibility and Bioactivity of the Surfaces

To assess cytocompatibility, the metabolic activity of MC3T3-E1 preosteoblastic cells was evaluated after 3 and 7 days of culture (Figure 6A–A’). On day 3 (Figure 6A), cell viability was influenced by both surface treatment and substrate. For cpTi, no statistically significant differences were observed between untreated and PEO-treated samples, indicating comparable metabolic activity; in contrast, the cpTi-SLA group showed significantly lower viability. For Ti-6Al-4V, the untreated surface exhibited the highest metabolic activity, whereas SLA treatment led to a statistically significant reduction. The PEO-treated Ti-6Al-4V group presented intermediate values, with no statistically significant difference compared to the untreated condition. On day 7 (Figure 6A’), metabolic activity values were generally higher than those observed at day 3, consistent with progressive cell proliferation over time. All experimental groups showed significantly higher viability than the negative control, indicating the absence of cytotoxic effects. Among cpTi samples, SLA- and PEO-treated surfaces exhibited no statistically significant differences, suggesting a convergence of cellular response over time. In contrast, for Ti-6Al-4V, untreated and PEO-treated groups remained statistically comparable, whereas SLA-treated surfaces consistently exhibited lower metabolic activity.

**Figure 6:**
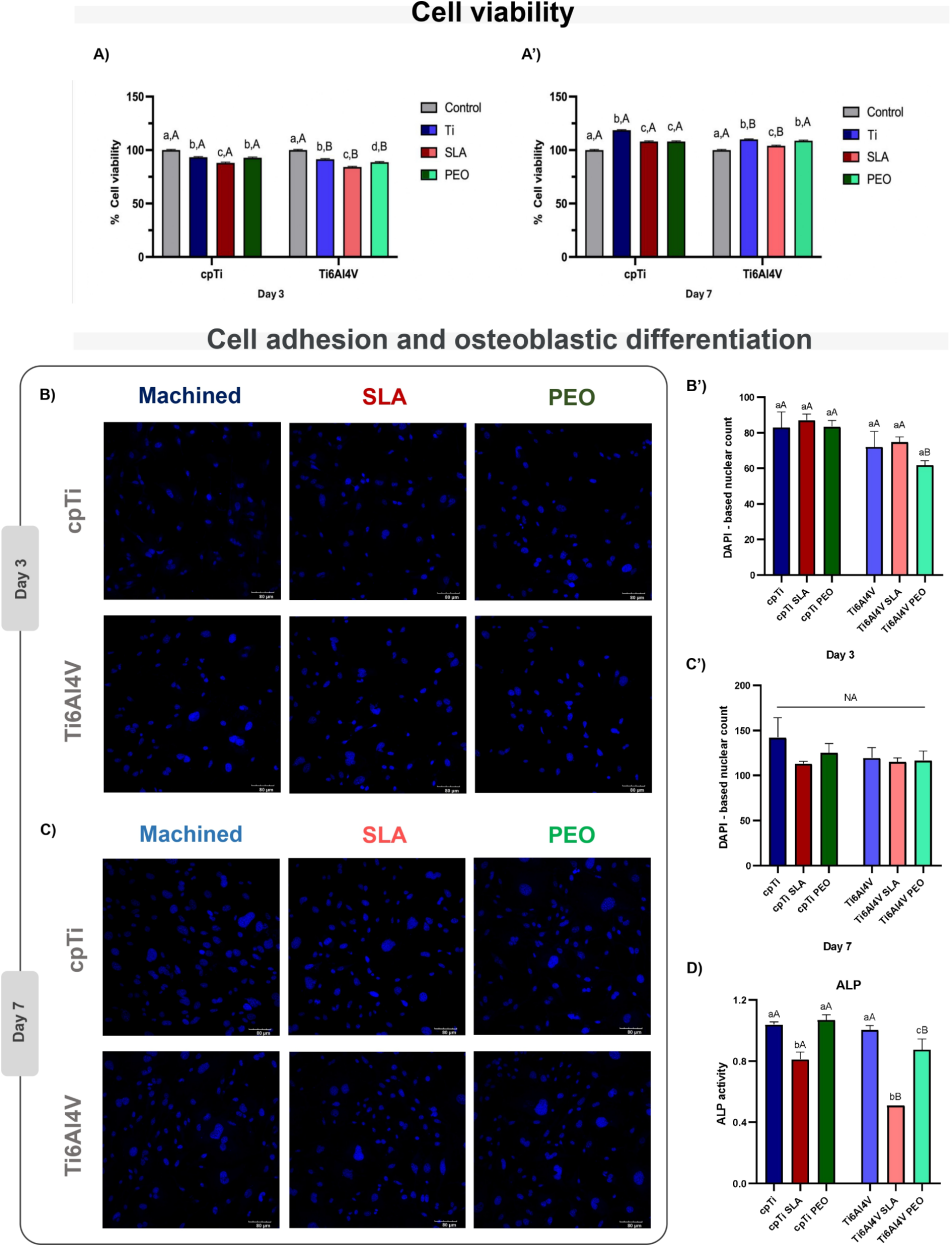
Cytocompatibility assay (*n* = 3/group) and cell adhesion and proliferation (*n* = 3/group). (A, A’) Effects of surfaces on MC3T3-E1 cell metabolism at 3 and 7 days. (B, C) Nuclei (blue) staining of cells demonstrating morphological behavior. (B’,C’) Cell count by fluorescence at 3 and 7 days based on cell nuclear count after DAPI staining. Different lowercase letters indicate statistically significant differences among surface treatments. Different uppercase letters indicate statistically significant differences between materials (*p* < 0.05, Tukey’s test). (D) ALP activity quantification at 7 days to evaluate osteoblastic differentiation (*n* = 3/group).

Overall, cpTi-based samples tended to exhibit higher metabolic activity than Ti-6Al-4V under similar conditions, although this effect depended on the specific surface treatment and statistical comparison. Importantly, although previous studies have frequently reported enhanced cytocompatibility on PEO-treated titanium surfaces compared with machined controls,^1–3^ this effect is not universal and appears to be highly dependent on the specific physicochemical characteristics generated by the plasma oxidation process. Parameters such as oxide thickness, pore morphology, roughness profile, incorporated ionic species, and substrate composition may substantially influence early cell–surface interactions.^18,43^ In the present study, the absence of a consistent metabolic advantage of PEO-treated samples over untreated cpTi suggests that the surface features produced under the adopted conditions were not sufficient to enhance early MC3T3-E1 metabolic activity. Conversely, the untreated machined cpTi surface may have provided a more homogeneous and less topographically complex interface, favoring initial cell attachment and metabolic performance. Importantly, these findings do not necessarily preclude potential benefits of PEO for other biological outcomes, since early metabolic activity represents only one aspect of cytocompatibility and may not fully predict later events such as osteogenic differentiation.

Cell adhesion and proliferation were further evaluated by DAPI nuclear staining, combining qualitative fluorescence imaging with quantitative nuclei counting to characterize the cell–material interface (Figure 6B–C’). Overall, the representative micrographs were consistent with the quantitative data, allowing visualization of differences in cell distribution and density across substrates and surface treatments. At day 3 (Figure 6B–B’), early differences in cell adhesion were evident. Quantitative analysis showed that, for cpTi, no significant differences were detected among machined, SLA, and PEO surfaces, indicating a comparable number of adherent cells across treatments. For Ti-6Al-4V, the PEO-treated group showed the lowest nuclei count; although no significant differences were observed among surface treatments within this substrate, PEO-treated cpTi exhibited a significantly higher nuclei density than PEO-treated Ti-6Al-4V. Overall, these findings were consistent with the fluorescence micrographs, which showed a relatively homogeneous cell distribution across groups and nuclear density. At day 7 (Figure 6C–C’), nuclei count increased in all groups, indicating progressive cell proliferation during the culture period. At this time point, no statistically significant differences were observed among substrates or surface treatments, demonstrating that the early differences detected at day 3 were attenuated over time. This pattern was also supported by the fluorescence images, which showed a denser, more uniformly distributed cell population across all surfaces. These findings are consistent with the metabolic activity data and reinforce the overall cytocompatibility of the tested surfaces.

ALP activity was evaluated at day 7 as an early marker of osteoblastic differentiation, given its role in providing phosphate for hydroxyapatite formation during mineralization (Figure 6D). Surface treatment significantly influenced ALP activity, although this effect was clearly substrate-dependent. For cpTi, the untreated and PEO-treated groups showed statistically similar ALP activity, and both were significantly higher than that of the SLA-treated surface. In contrast, for Ti-6Al-4V, the untreated surface exhibited the highest ALP activity, followed by the PEO-treated group, whereas SLA treatment resulted in the lowest values. These findings indicate that PEO did not uniformly enhance osteoblastic differentiation relative to the untreated condition; rather, its effect depended on the underlying substrate. The distinct ALP response observed between cpTi-PEO and Ti-6Al-4V-PEO may be associated with differences in the surface topography and coating architecture generated by the PEO process on each substrate.

Regarding substrate-related effects, cpTi and Ti-6Al-4V exhibited comparable ALP activity under untreated conditions; however, after surface modification, cpTi-based groups showed higher ALP activity than their Ti-6Al-4V counterparts for both SLA and PEO. Notably, cpTi and cpTi-PEO exhibited the highest ALP levels, whereas Ti-6Al-4V-SLA presented the lowest values. When interpreted together with the viability and DAPI results, these findings suggest that PEO did not necessarily increase early metabolic activity but maintained osteoblastic differentiation more favorably than SLA, particularly on cpTi. Therefore, the biological effect of PEO appears to result from an interaction between surface treatment and substrate composition rather than from the treatment alone.

The bioactivity of the treated surfaces was assessed by evaluating hydroxyapatite (HAp) nucleation growth after 21 days of immersion in SBF, as shown in Figures 7A, A’. Morphological differences in HAp deposition were clearly observed in SEM micrographs (Figure 7A’), which revealed distinct surface-dependent patterns. SLA-treated samples exhibited nanoflake-like structures, regardless of the substrate. In contrast, the PEO groups presented predominantly hexagonal morphologies and microsphere-like features. Interestingly, machined surfaces exhibited divergent behaviors: whereas the Ti-6Al-4V alloy favored the formation of nanoflakes, cpTi surfaces exhibited microsphere-like structures.

**Figure 7:**
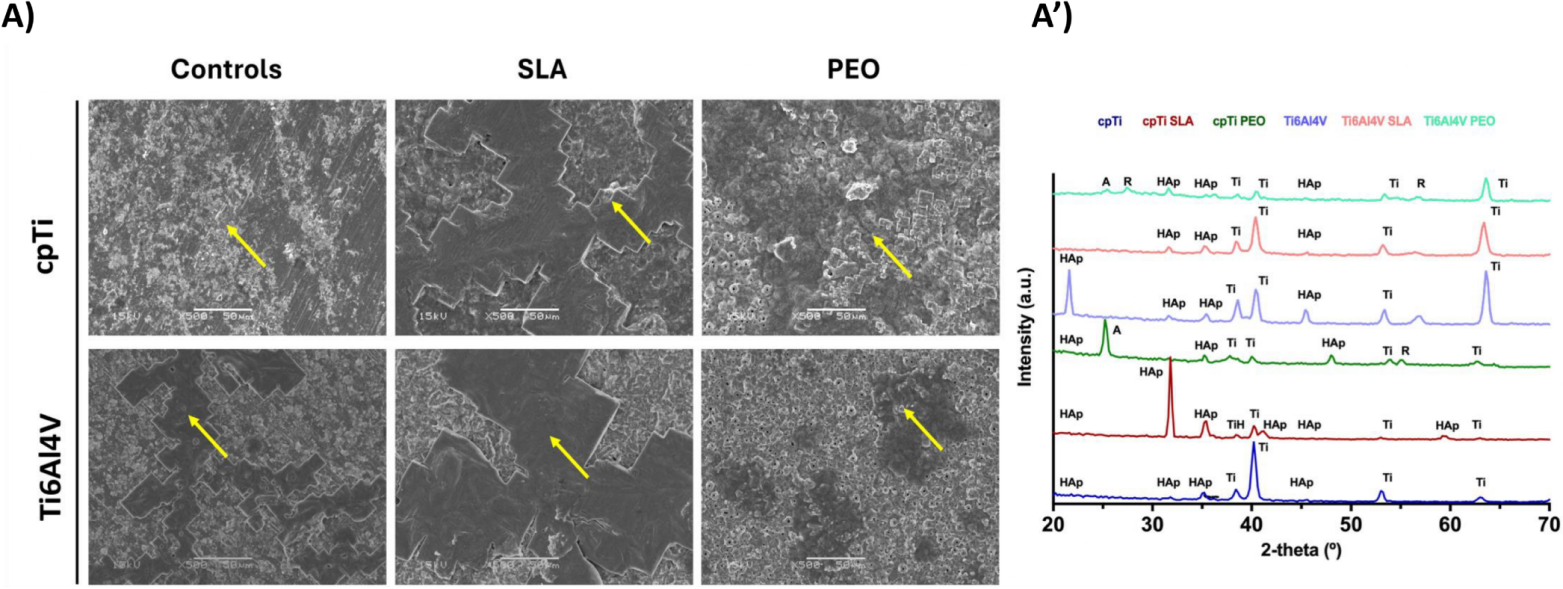
For the bioactivity assay, samples were immersed in SBF for 21 days to induce HAp formation on their surfaces. (A) SEM micrographs (500× magnification) were obtained to evaluate HAp morphology. (A’) XRD analysis was performed to confirm HAp formation (Ti = titanium, R = rutile, and HAp = hydroxyapatite). Different lowercase letters indicate statistically significant differences among surface treatments. Different uppercase letters indicate statistically significant differences between materials (*p* < 0.05, Tukey’s test).

Complementary information was provided by XRD analyses (Figure 7A). Phase identification was carried out by comparing the experimental XRD patterns with JCPDS reference files. The peaks observed at 2θ ≈ 25.9°, 31.8°, 32.2°, and 32.9° were assigned to hydroxyapatite (HAp, JCPDS No. 09–0432), corresponding to the characteristic triplet in the 31–33° range. In addition, low-intensity diffraction peaks observed at approximately 46° and 60° were attributed to secondary reflections of hydroxyapatite, namely the (222) and high-index planes, respectively. The low intensity and broadened peaks of these reflections are consistent with the formation of a poorly crystalline, biomimetic apatite layer following SBF immersion. The reflections at 2θ ≈ 35.1°, 38.4°, and 40.2° correspond to α-Ti (JCPDS No. 44–1294), originating from the metallic substrate. Additional peaks at 2θ ≈ 27.4° and 36.1°, along with weaker reflections in the 50–60° range, indicate the presence of rutile TiO_2_ (JCPDS No. 21–1276), particularly in the PEO-treated samples, as a result of the high local temperatures generated during the microarc oxidation process. Low-intensity peaks associated with δ-TiH_2_ (JCPDS No. 09–0371) were observed mainly on the SLA-treated surfaces, likely related to hydrogen uptake during the acid etching step. The diffraction patterns also suggested the presence of calcium and phosphorus agglomerates, likely associated with electrostatic interactions at the surface.^34^ These findings support the effectiveness of both surface treatments in promoting HAp formation at levels comparable to those observed in machined surfaces. Moreover, comparative analysis between substrates showed consistent apatite layer development, indicating that the bioactive response was maintained regardless of the base material. Notably, PEO-treated surfaces exhibited bioactivity comparable to that of SLA-treated ones, further reinforcing PEO as a promising strategy for surface modification in titanium-based implants.

Taken together, the cellular and HAp formation results provide complementary evidence of the biological performance of the modified surfaces. While both SLA and PEO treatments were effective in promoting apatite nucleation after SBF immersion, indicating comparable *in vitro* bioactivity, the significantly higher ALP activity observed for PEO-treated samples suggests a more pronounced ability to induce osteogenic differentiation at the cellular level when compared to SLA.

## 4 Discussion

To address the need for dental implants with superior mechanical integrity and enhanced biological performance, this study evaluated the effects of surface treatments on cpTi and Ti-6Al-4V substrates, focusing on their physicochemical, mechanical, electrochemical, and bioactive properties. While both materials are well established in implant dentistry due to their biocompatibility, their surface behavior under functional conditions remains a critical determinant of long-term success.^10^ Long-term outcomes depend on an optimized biomaterial–tissue interface that supports the biological events required for osseointegration.^45–48^ The widely adopted SLA treatment demonstrated a suitable bioactive response; however, limitations, including evolving corrosion and wear resistance, were observed. In contrast, PEO has emerged as a promising alternative, enabling the formation of oxide layers with tailored microtopography, increased hardness, and enhanced electrochemical stability.^49,50^ Our findings confirm that PEO-treated surfaces outperformed SLA and control groups across multiple parameters, regardless of the metallic substrate, supporting their potential to optimize the biological-mechanical interface of dental implants.

The microstructural properties of the substrate play a pivotal role in determining the morphology and characteristics of the resulting surface treatments, as evidenced by SEM analyses. Substrate microstructure influences not only the response to surface modification but also the uniformity of the formed layer and its behavior under mechanical stress.^18,29^ In this context, the grain size and intrinsic hardness of the Ti-6Al-4V alloy affect both its polishing behavior and the adhesion and stability of the surface treatments. These features impact the alloy’s resistance to acid etching and to the microdischarges generated during anodic layer formation, as well as its resistance to wear and corrosion.^42^ Previous studies^51,52^ have shown that substrate composition directly influences the surface topography resulting from acid etching in SLA-treated samples. Our findings support this notion, showing that treatments applied to cpTi produced more pronounced surface features than those used to Ti-6Al-4V, with greater peak-to-valley amplitudes. This topographic variation may influence biological responses by modulating the implant–tissue interface and promoting distinct osseointegration mechanisms.

According to the XRD patterns, titanium hydrides were detected exclusively in SLA-treated cpTi, likely resulting from the interaction between Ti atoms and free hydrogen ions in the acidic solution.^52,53^ Although their formation appears localized, these brittle phases may compromise the long-term mechanical and chemical stability of the implant due to their susceptibility to fracture under cyclic loading.^42^ In contrast, PEO-treated surfaces exhibited well-defined anatase and rutile crystalline phases, formed through electrochemical oxidation involving Ti and O species from the electrolyte and associated with the incorporation of calcium and phosphorus.^35,54^ The coexistence of these crystalline phases enhances both mechanical resistance and bioactivity, key factors for ensuring long-term implant performance.^34,50^

Regarding surface roughness and wettability, both crucial to implant–tissue interactions, these properties varied according to the type of treatment applied. SLA-treated surfaces exhibited significantly higher roughness due to the synergistic effect of mechanical abrasion and acid etching, which generated a complex hierarchical topography with sharp peaks and deep valleys.^13,42,55^ In contrast, PEO-treated surfaces exhibited significantly lower roughness than SLA, attributable to the formation of a more uniform and compact oxide layer resulting from localized melting and electrochemical ion incorporation.^34^ Wettability results reflected the combined influence of surface texture and chemistry on contact angle values. The Ti-6Al-4V control group exhibited higher hydrophilicity, likely due to its smoother surface and greater surface free energy. Although an inverse relationship between surface roughness and contact angle is frequently reported, wettability is governed by the synergistic effects of surface topography and surface chemistry.^17,35^ In the present study, SLA-treated surfaces exhibited increased roughness accompanied by higher contact angles and lower surface free energy. This behavior can be explained by the formation of sharp microfeatures and deep valleys during acid etching, which favor air entrapment beneath the liquid droplet, leading to a Cassie–Baxter wetting regime rather than complete wetting.^16,55,56^ In addition, the chemical modifications induced by the acid treatment may reduce the density of surface hydroxyl groups, decreasing the polar component of surface free energy. Consequently, despite the higher roughness, the combined effects of air trapping and reduced surface energy result in increased contact angles on SLA-treated surfaces.^13,57^

Nevertheless, all groups featured hydroxylated TiO_2_ layers that formed hydrogen bonds with water molecules, thereby enhancing surface wettability.^57,58^ Notably, Ti-6Al-4V-PEO exhibited slightly improved wettability compared to cpTi-PEO, possibly due to subtle differences in micropore architecture.^32^ These findings underscore that even slight modifications in surface properties, such as wettability, roughness, and chemical composition, can critically modulate biological responses, influencing protein adsorption, bioactivity, and implant osseointegration.^59^

Furthermore, mechanical properties are critical to ensuring implant durability under functional loading conditions. As expected, Ti-6Al-4V exhibited significantly higher hardness than cpTi across all surface treatments, a result attributed to its stable biphasic (α + β) microstructure at room temperature. This configuration confers superior tensile strength, yield strength, and surface hardness, thereby enhancing its mechanical performance relative to cpTi.^7,60^ The PEO-treated surfaces exhibited higher hardness than the SLA-treated groups, regardless of the metallic substrate. This enhancement is likely associated with the formation of a thicker, denser oxide layer that incorporates complex oxides and the rutile phase, which contribute to improved surface strength.^21,61,62^ By comparison, SLA-treated surfaces showed only subtle, nonsignificant changes in friction and mass loss, a behavior that may be linked to a relatively less stable and more heterogeneous surface film produced by the SLA process,^63^ which can undergo localized removal under sliding without necessarily causing a consistent, measurable reduction in hardness at the scale of the Vickers microhardness tests.^64–66^ Interestingly, the coexistence of both phases and the refined grain structure of Ti-6Al-4V enhanced the coatings’ resistance to wear and plastic deformation, thereby minimizing the wear area and surface degradation observed after tribological testing.^29,67,68^ During testing, the PEO group on Ti-6Al-4V exhibited an increased coefficient of friction, which can be viewed positively in a clinical context. A higher friction coefficient between the implant and bone has been associated with improved primary stability, thereby reducing the risk of micromotion during early osseointegration and promoting implant success.^34^ Together, these findings highlight the importance of tribological performance as a complementary factor to bioactivity and corrosion resistance in the development of implant surfaces. Notably, the PEO treatment demonstrated a synergistic combination of hardness and wear resistance, reinforcing its potential to enhance the mechanical reliability of dental implants subjected to occlusal forces.^32,34^

In addition to mechanical performance, corrosion kinetics are crucial in metallic implants, as degradation processes can compromise structural integrity and lead to ion release, potentially triggering chronic inflammation and implant failure.^10,69^ During a 1-h immersion in SBF, all groups formed a stable oxide layer, indicating early surface passivation. PEO-treated samples exhibited more electropositive OCP values, suggesting lower corrosion susceptibility due to denser, more stable oxide layers.^42^ These surfaces also exhibited higher impedance at low frequencies, a greater phase angle at high frequencies, and a larger Nyquist arc magnitude than the control and SLA groups. Notably, cpTi-PEO presented slightly higher phase angles than Ti-6Al-4V-PEO, indicating superior electrochemical stability.^4,32^ SEM and CLSM revealed that Ti-6Al-4V-PEO formed a compact layer with a narrow pore spacing, allowing greater electrolyte penetration and reducing barrier efficacy.

In contrast, cpTi-PEO exhibited a thicker, denser layer with wider interpore spacing, improving electrolyte resistance and corrosion protection.^70^ These morphological differences directly influenced corrosion resistance, as confirmed by higher total polarization resistance (R_ptot_) in the PEO groups. This behavior reflects the formation of compact, adherent TiO_2_ layers enriched with rutile and doped with calcium and phosphorus, which enhances their protective capacity.^21,32^ SLA and control groups showed lower R_ptot_ values, indicating increased ion infiltration and oxide degradation.^71^ Although substrate composition played a minor role, cpTi-PEO showed slightly better performance, likely due to the lower hardness of cpTi, which may favor more effective fusion and oxide formation during the PEO treatment process.^72^

In Figure 5, the PEO–CaP-treated conditions (cpTi-PEO and Ti-6Al-4V-PEO, represented by the green curves) were processed using identical PEO parameters but on different substrates and prior surface states (commercially pure Ti vs Ti-6Al-4V). This results in subtle differences in coating morphology and dielectric behavior, as confirmed by SEM and CLSM: Ti-6Al-4V-PEO develops a more compact layer with narrower interpore spacing, favoring electrolyte permeation, whereas cpTi-PEO forms a thicker and denser oxide with wider interpore spacing (with an approximate coating thickness of 5–10 μm, as previously reported by this research group.^43,70^ These microstructural variations slightly affect the effective capacitance and charge-transfer resistance of the inner barrier and outer porous layers, resulting in slight differences in the shape and magnitude of the green curves in the Bode, Nyquist, and phase-angle plots.^32,71,72^ Nevertheless, the overall trend is consistent and robust: both PEO groups exhibit more electropositive OCP values, the highest impedance at low frequencies, larger Nyquist semicircles, and higher phase angles than their respective control and SLA counterparts. This indicates that the electrochemical response is primarily governed by the presence of the PEO–CaP bilayer. In contrast, the substrate (cpTi vs Ti-6Al-4V) plays only a secondary role in the fine details of the EIS response.^32^

Although no statistically significant differences were detected in corrosion potential and corrosion rate among the groups in the potentiodynamic polarization tests, this finding is consistent with the formation of a stable passive film on all surfaces and with the relatively narrow potential window used. Although cpTiSLA exhibited the lowest polarization resistance among the tested surfaces, its potentiodynamic polarization curve still showed a relatively stable behavior, with no evidence of abrupt current spikes or pitting-like events within the potential range studied (Figure 5H). This indicates that a passive-like film is present on the SLA-treated surface. Still, its protective efficacy is lower than that of the PEO-CaP coatings, resulting in a higher corrosion current density and a lower Rp. The macroscopic shape of the curve, therefore, remains smooth, while the electrochemical parameters reveal a less resistant passive state.^73–75^ The shift in the corrosion potential and the reduction in Rp for cpTiSLA can be rationalized by considering both increased surface roughness and modified surface chemistry. The SLA process yields a markedly higher real surface area with numerous microasperities and defects, thereby increasing the density of active anodic and cathodic sites and facilitating electrochemical reactions.^76,77^ In addition, changes in the oxide film (thickness, composition, and defect density) and potential surface contamination may increase the passive layer’s susceptibility to dissolution. Together, these effects result in a more negative E_corr_ and a lower Rp for cpTiSLA. In contrast, the thicker, chemically distinct PEO-CaP coatings are more effective barrier layers, shifting E_corr_ to more noble values and increasing Rp.^75^ Taken together, these observations suggest that SLA and PEO do not substantially alter the basic passivation behavior reflected in the polarization curves, but PEO-CaP clearly enhances the surface’s barrier and protective character, as captured by EIS.

Finally, HAp, a crystalline calcium phosphate, is widely recognized as a key bioactive agent in promoting osseointegration. The formation of bioactive ceramic HAp layers on the surface of implantable materials plays a pivotal role in enhancing cellular adhesion and bone anchorage.^78,79^ Its spontaneous nucleation on titanium surfaces is driven by the presence of hydroxylated TiO_2_ layers, which attract Ca^2+^ and PO_4_^3–^ ions through electrostatic interactions, as explained by Pauling’s electroneutrality principle.^25,80^ The microtopography generated by PEO treatment, characterized by high roughness and porosity, further enhances this process by promoting heterogeneous nucleation and irregular HAp deposition.^23,81,82^ These HA-like deposits not only indicate potent bioactivity but also create a favorable environment for cell adhesion, proliferation, and osteogenic differentiation, reinforcing the clinical relevance of such surface modifications.^34,78^

The present study demonstrates that early cytocompatibility outcomes (metabolic activity and initial adhesion/proliferation) and early osteogenic function (alkaline phosphatase, ALP) are not necessarily coupled. The direction of the cellular response depends on how each surface condition integrates topography, wettability/surface free energy, and surface chemistry/oxide characteristics. In a clinical scenario, immediately after implant placement, the surface comes into contact with body fluids and proteins, forming a conditioning layer that influences subsequent cell–material interactions.^43^ In this context, the early adhesion of osteoblasts to implant surfaces plays a decisive role in guiding cell differentiation, which is fundamental to the successful osseointegration process.^83,84^ In addition to demonstrated cytocompatibility, the temporal evolution of cell responses highlights substrate- and treatment-dependent effects.^85,86^ The polished surfaces, characterized by a relatively homogeneous topography with polishing grooves, tend to exhibit better initial performance. Increasing the roughness of Ti-6Al-4V through abrasive treatments can reduce the adhesion and activity of MC3T3-E1 compared to polished states.^87^

In contrast, although the SLA-type treatment significantly increased roughness (Ra) and produced the expected microrough morphology (microdepressions and sharp features), it also increased the contact angle and reduced surface free energy, indicating a less-wettable interface. This combination can impair early protein adsorption dynamics and cell spreading, contributing to the observed reduction in metabolic activity, particularly on Ti-6Al-4V, where the SLA group maintained lower viability even at day 7.^88–90^ Taken together, these findings support that the inferior performance of SLA reflects the combined effects of microtopography, wettability, and treatment-related surface chemical modifications.

The distinct ALP responses observed for cpTi-PEO and Ti-6Al-4V-PEO indicate that the biological effect of PEO depended not only on the treatment itself but also on the specific coating architecture generated on each substrate. Variations in surface topography, particularly in the size and distribution of peaks and valleys observed by SEM, may have modulated cell–surface interactions and, consequently, influenced osteoblastic differentiation.^32^ In parallel, PEO generated a uniform porous layer enriched with Ca and P and containing crystalline TiO_2_ phases (anatase and rutile), features commonly associated with improved osteogenic behavior. Although this treatment did not significantly enhance early metabolic activity relative to untreated surfaces, it favored ALP activity at day 7, indicating that its main contribution was not to increase initial proliferation but rather to direct cells toward a more differentiated osteoblastic phenotype. This interpretation is consistent with previous studies reporting enhanced differentiation and mineralization on Ca/P-containing PEO-treated Ti-6Al-4V, with no evidence of cytotoxicity.^91–93^ Moreover, the marked reduction in the quantitative V signal after PEO on Ti-6Al-4V suggests a lower direct contribution of alloying elements at the cell–material interface, which may partly explain the more favorable osteogenic response of PEO compared with SLA on the alloy. Taken together, these findings indicate that the osteogenic performance of PEO-treated surfaces cannot be generalized across titanium substrates, as it is governed by the combined influence of topography, oxide chemistry, phase composition, porosity, and substrate-dependent coating formation.

Complementing these cellular responses, the ability of the surfaces to promote mineral deposition is also essential, with HAp being widely recognized as a key indicator of bioactivity.^34^ The spontaneous formation of HA-like deposits across all coated groups (regardless of the metallic substrate) is a promising outcome, as these deposits not only reflect strong bioactive potential but also foster a favorable environment for cell adhesion, proliferation, and osteogenic differentiation, thereby reinforcing the clinical relevance of such surface modifications.^78,94^ Morphologically, HAp deposition varied depending on surface topography: nanoscale granular particles predominated on polished cpTi surfaces, whereas microsphere-like structures were observed on PEO-treated groups. These differences suggest that the higher roughness and irregular microstructure of anodized surfaces enhance heterogeneous nucleation and promote uneven HAp growth, which may further stimulate osteogenic responses under physiological conditions.

## 5 Conclusion

Surface treatments by PEO and SLA exhibited distinct yet complementary effects on cpTi and Ti-6Al-4V, offering strategies to optimize dental implant performance. Although all surfaces exhibited bioactivity, the PEO coating formed a porous oxide layer enriched in calcium and phosphorus, which significantly enhanced functional properties. From a mechanical perspective, PEO-treated surfaces exhibited increased hardness and improved resistance to surface damage, which may contribute to greater wear resistance under functional loading. In addition, from an electrochemical standpoint, PEO surfaces exhibited superior corrosion resistance, likely due to the formation of a thicker, more stable oxide layer that serves as an effective barrier to ionic exchange in aggressive environments.

From a biological perspective, PEO did not consistently enhance early metabolic activity relative to untreated surfaces; however, it maintained cytocompatibility and supported osteoblastic differentiation more favorably than SLA, as evidenced by ALP results. Overall, while SLA remains a clinically established surface treatment, PEO provided a more comprehensive improvement by simultaneously enhancing mechanical performance, electrochemical stability, and osteogenic potential. Future studies should therefore explore hybrid or optimized surface-engineering strategies capable of combining the clinically validated features of SLA with the bioactive and protective benefits of PEO to further improve long-term implant performance.

## Supplementary Material



## Notes

Additional methodological details on tribological testing, electrochemical behavior assessment, and biological performance analysis, including preosteoblastic cell culture and cell viability assay (PDF)

## #

Department of Prosthodontics and Periodontology, Bauru School of Dentistry (FOB/USP), University of São Paulo, Bauru 17012–901, São Paulo, Brazil
