# Supplementary material for: Surface Modifications of cpTi and Ti-6Al‑4V: The Synergistic Potential of PEO-CaP for Optimized Dental Implant Performance
Source: ACS Omega. 2026 Jul 6;11(28):41287–303. doi: 10.1021/acsomega.5c12236 (PMC13393044; doi:10.1021/acsomega.5c12236)
Supplement: Supplementary file 1 [file ao5c12236_si_001.pdf]

Supplementary data

Surface Modifications of cpTi and Ti-6Al-4V: The Synergistic Potential of PEO-CaP for Optimized Dental Implant Performance

Júlia M. T. Teodoro[a,1], Maria H. R. Borges[a,1], Raphael C. Costa[b], Jairo M. Cordeiro[c], Elidiane Rangel[d], Nilson C. Da Cruz[d], Carlos A. Fortulan[e], Bruna E. Nagay[f], Valentim A. R. Barão[a*]

[a] Universidade Estadual de Campinas (UNICAMP), Piracicaba Dental School, Department of Prosthodontics and Periodontology, Piracicaba, São Paulo 13414-903, Brazil.

[b] Alfenas Federal University (UNIFAL), School of Dentistry, Alfenas, Minas Gerais 37130-001, Brazil.

[c] University Center of Associated Teaching Faculties (UNIFAE), São João da Boa Vista, São Paulo, 13870-377, Brazil

[d] São Paulo State University (UNESP), Institute of Science and Technology, Laboratory of Technological Plasmas, Sorocaba, São Paulo 18087-180, Brazil

[e] University of São Paulo (USP), Department of Mechanical Engineering, São Carlos, São Paulo 13566-590, Brazil.

[f] University of Sao Paulo - Bauru School of Dentistry (FOB-USP), Department of Prosthodontics and Periodontology, Bauru, São Paulo, 17012-901, Brazil.

*** Corresponding author at:**

Universidade Estadual de Campinas (UNICAMP), Piracicaba Dental School, Department of Prosthodontics and Periodontology, Av. Limeira, 901, Piracicaba, São Paulo 13414-903, Brazil.

E-mail address: vbarao@unicamp.br (V.A.R. Barão).

[1] These authors share the first authorship

Experimental Details

Tribological test

The friction coefficient was determined using a custom-built pin-on-disk tribometer (Faculty of Mechanical Engineering, University of São Paulo, São Carlos, Brazil) [39]. Tests were performed with a Zr sphere (Y-TPZ; ø = 5 mm) sliding against the sample surface immersed in simulated body fluid (SBF) under a vertical load of 5 N, track diameter of 7 mm, sliding speed of 0.01 m/s, and duration of 100 s. Friction evolution and average coefficient values were recorded with LabView software (National Instruments, São Paulo, Brazil). Mass loss (mg) was calculated by weighing each disc before and after testing using an analytical balance (AUY-UNIBLOC, Shimadzu Corporation, Kyoto, Japan) [34]. After tribological testing, wear scars were analyzed by SEM (JEOL JSM-6010 LA, Peabody, MA, USA). Wear area was determined using an optical microscope (VMM-100-BT; Walter UHL, Asslar, Germany) coupled with a digital camera (KC-512NT; Kodo BR Eletronica Ltd., São Paulo, Brazil) and an analyzer unit (QC 220-HH Quadra Check 200; Metronics Inc., Bedford, MA, USA). Total wear surface was calculated using the formula $2\pi rd + \pi d^2$, where r is the inner disc radius and d is the wear track width. Measurements were performed by a calibrated examiner [39].

Electrochemical behavior

Electrochemical tests were performed to assess the corrosion stability of the surfaces in simulated body fluid (SBF; 10 mL; 37 °C), following previous protocols. A three-electrode cell, connected to a potentiostat (Interface 1000, Gamry Instruments, Warminster, PA, USA), was used to perform open-circuit potential (OCP) measurements, electrochemical impedance spectroscopy (EIS), and potentiodynamic polarization. Samples were first cathodically polarized at −0.9 V (vs. reference electrode) to reduce and standardize the oxide layer. OCP was monitored for 1 hour to evaluate the material's potential and system stabilization. EIS was carried out over a frequency range of 100 kHz to 5 mHz, and the data were analyzed using Echem Analyst (Gamry Instruments) with the appropriate equivalent circuits. Nyquist plots, impedance modulus (|Z|), and phase angle were obtained from real

(Z′) and imaginary (Z″) components. Potentiodynamic polarization was performed from −0.8 V to 1.8 V at a scan rate of 2 mV/s [30]. Corrosion parameters—including corrosion potential (E_{corr}), Tafel slopes (βcathodic, βanodic), and corrosion rate—were determined by Tafel extrapolation using Echem Analyst. Surface area values (in cm^2) considered for analysis were: cpTi = 3.40; cpTi-SLA = 8.23; cpTi-PEO = 5.72; Ti6Al4V = 3.31; Ti6Al4V-SLA = 3.48; Ti6Al4V-PEO = 5.29.

Pre-osteoblastic cell culture and cell viability

To evaluate cytocompatibility, mouse calvaria-derived pre-osteoblastic MC3T3-E1 cells (ATCC CRL-2594; Banco de Células do Rio de Janeiro) were cultured directly on the sample surfaces. Cells were maintained in α-MEM (Gibco, Life Technologies, USA) supplemented with 10% fetal bovine serum (FBS; Gibco, Grand Island, NY, USA), 100 U/mL penicillin, and 100 μg/mL streptomycin, under standard conditions (37 °C, 5% CO_2). When cultures reached ~80% confluence, cells were detached with trypsin-EDTA (Gibco), resuspended in supplemented medium, and seeded onto the titanium disks at 1×10^4 cells/well in 24-well plates. Cultures were maintained for 24 h to allow monolayer formation, following ISO 10993-5 guidelines. Medium was replaced every other day throughout the experiment. Later, cell metabolic activity was assessed by the AlamarBlue assay. At days 3 and 7, culture medium was replaced with 500 μL of fresh medium containing 10% alamarBlue reagent (Invitrogen, Carlsbad, CA, USA). After 4 h of incubation at 37 °C, 100 μL of supernatant from each well was transferred to a 96-well plate, and absorbance was measured at 570 and 600 nm using a microplate reader (Multiskan, Thermo Scientific, Vantaa, Finland) [43].
